# Expression of ERK1/2, p38, and JNK in Normal Kidney Development and CAKUT

**DOI:** 10.3390/medicina62030549

**Published:** 2026-03-16

**Authors:** Ivona Letica, Petar Todorović, Patricija Bajt, Nikola Pavlović, Nela Kelam, Marjana Jerković Raguž, Ivanka Mikulić, Ludvig Letica, Sandra Kostić, Katarina Vukojević, Anita Racetin

**Affiliations:** 1 Department of Pediatric Nephrology, University Clinical Hospital Mostar, 88000 Mostar, Bosnia and Herzegovina; ivonaletica1@gmail.com; 2 School of Medicine, University of Mostar, 88000 Mostar, Bosnia and Herzegovina; marjanajerkovic@yahoo.co.uk (M.J.R.); katarina.vukojevic@mefst.hr (K.V.); 3 Department of Anatomy, Histology and Embryology, School of Medicine, University of Split, 21000 Split, Croatia; petar.todorovic@mefst.hr (P.T.); patricija.bajt@mefst.hr (P.B.); nikola.pavlovic@mefst.hr (N.P.); nela.kelam@mefst.hr (N.K.); sandra.kostic@mefst.hr (S.K.); 4 Department of Neonatology, University Clinical Hospital Mostar, 88000 Mostar, Bosnia and Herzegovina; 5 Department of Laboratory Diagnostics, University Clinical Hospital Mostar, 88000 Mostar, Bosnia and Herzegovina; ivankacolak@yahoo.com; 6 Faculty of Pharmacy, 88000 Mostar, Bosnia and Herzegovina; 7 Department of Surgery, University Clinical Hospital Mostar, 88000 Mostar, Bosnia and Herzegovina; ludvigletica@gmail.com; 8 Center for Translational Research in Biomedicine, School of Medicine, University of Split, 21000 Split, Croatia; 9 Mediterranean Institute for Life Sciences, University of Split, Meštrovićevo Šetalište 45, 21000 Split, Croatia

**Keywords:** p38, JNK, ERK1/2, CAKUT, fetal kidney development

## Abstract

*Background and Objectives:* Mitogen-activated protein kinases (p38, JNK, ERK1/2) regulate key cellular processes essential for kidney development. Disruptions in these signaling pathways can lead to congenital anomalies of the kidney and urinary tract (CAKUT), a major cause of pediatric kidney disease. This study investigates and compares the expression of these molecules in normal fetal kidneys and CAKUT-affected tissues. *Materials and Methods:* Forty-three human fetal kidney samples, including controls and specimens with horseshoe, hypoplastic, and dysplastic kidneys, were analyzed across developmental phases 2–4 using immunofluorescence. Quantitative image analysis and statistical comparisons were performed between developmental stages and phenotypes. *Results:* ERK1/2 expression increased during late development in control kidneys but was significantly reduced in hypoplastic kidneys. p38 showed phase-dependent alterations, with early upregulation in dysplastic kidneys and late elevation in horseshoe kidneys. JNK exhibited significant phase-dependent upregulation in horseshoe kidneys. P38 displayed dynamic expression associated with nephron maturation. *Conclusions:* MAPK pathways show distinct developmental and phenotype-specific expression patterns in human fetal kidneys. These differences reflect divergent pathogenic mechanisms in CAKUT and may support improved molecular characterization of congenital renal anomalies.

## 1. Introduction

The initiation of human fetal renal development occurs in early gestation, marked by the formation of the pronephros, a transient, nonfunctional kidney precursor [1]. This is followed by the mesonephros, which functions briefly during the first trimester before involution [1,2]. The permanent adult kidney, known as the metanephros, initiates development approximately during the fifth week of gestation and subsequently undergoes intricate morphogenetic processes involving ureteric bud branching and specialization of the metanephric mesenchyme [2]. By 9–12 weeks, fetal kidneys become identifiable, with ongoing nephrogenesis continuing until approximately 32 weeks, resulting in the formation of millions of nephrons required for postnatal renal function [1]. Congenital anomalies of the kidney and urinary tract (CAKUT) represent a spectrum of developmental defects arising from disruptions during these tightly regulated processes [3,4]. Such anomalies range from renal agenesis and dysplasia to abnormalities like hydronephrosis and cystic diseases, and they remain a leading cause of pediatric kidney failure [5].

The mitogen-activated protein kinase (MAPK) family consists of serine/threonine kinases organized into three principal subgroups: extracellular signal-regulated kinases (ERK), p38 MAPKs (p38), and c-Jun N-terminal kinases/stress-activated protein kinases (JNK) [6]. Our study focuses on elucidating the expression patterns of key signaling molecules p38, JNK, and ERK1/2, which are critical regulators of cellular proliferation, differentiation, and apoptosis during kidney development [7,8]. Understanding how these pathways are modulated in normal fetal kidneys and altered in CAKUT will provide insights into the molecular basis of renal developmental disorders [9].

p38 mitogen-activated protein kinase (p38 MAPK) is a critical signaling molecule involved in cellular responses to stress stimuli and regulation of cell differentiation, apoptosis, and homeostasis [10]. In kidney development, p38 MAPK is part of key signaling cascades that influence renal cell proliferation and differentiation [7]. Dysregulation of p38 MAPK signaling has been implicated in various kidney pathologies, including fibrosis and injury [11]. p38 is known to interact with pathways and genes fundamental to kidney morphogenesis [6]. Abnormalities in MAPK signaling pathways, including p38, can affect ureteric bud development and nephron formation, processes that when disrupted, contribute to CAKUT [12]. Furthermore, genetic studies indicate that pathways involving p38 may be modulated in CAKUT-related gene mutations, suggesting a contributory role of altered p38 MAPK activity in the etiology of these developmental kidney defects [13].

The c-Jun N-terminal kinase (JNK) signaling pathway plays a significant role in kidney development as well as in various renal diseases [14]. JNK is activated by stress stimuli and is involved in regulating cell proliferation, apoptosis, and inflammation [15,16]. JNK signaling plays a critical role in modulating the function of tubular epithelial cells and the development of nephrons [14]. Dysregulation of JNK has been linked to renal fibrosis and acute kidney injury, conditions that share mechanistic overlaps with developmental abnormalities [17]. In models of cystic kidney disease, inhibition of JNK signaling has been demonstrated to attenuate cyst growth, thereby underscoring the broader role of JNK in renal morphogenesis and disease progression [18].

The extracellular signal-regulated kinase 1/2 (ERK1/2) signaling pathway is crucial for nephrogenesis, particularly in the regulation of ureteric bud branching and nephron differentiation [19]. ERK1/2 activation affects cellular proliferation, differentiation, and morphogenesis of the developing kidney [20]. Inhibition of ERK1/2 disrupts nephron progenitor cell function and results in reduced nephron number, highlighting its essential role in renal organogenesis [20,21,22]. Furthermore, ERK1/2 signaling is involved in mediating responses to growth factors like epidermal growth factor (EGF), which are critical in renal development and homeostasis [19]. Aberrant ERK1/2 activity has also been implicated in renal pathologies such as fibrosis and cystic kidney diseases, conditions with potential developmental origins linked to CAKUT [23].

Understanding the molecular mechanisms underlying human fetal kidney development is critical for elucidating the origins of CAKUT, a leading cause of pediatric kidney disease. The MAPK pathways, including p38, JNK, and ERK1/2, are implicated in renal development, and their spatial and temporal activity in the human fetal kidney remains poorly characterized. Therefore, this study aims to map the spatial and temporal expression of p38, JNK, and ERK1/2 in the developing human fetal kidney and investigate their potential involvement in CAKUT pathogenesis.

## 2. Materials and Methods

### 2.1. Tissue Collection and Processing

This study utilized 43 human fetal kidney tissue samples (Table 1) obtained from cases of spontaneous pregnancy loss and elective terminations due to severe fetal abnormalities. The specimens were collected at the University Hospital in Split, specifically from the Department of Pathology. Tissue processing adhered to the principles outlined in the Declaration of Helsinki and was approved by the Ethical Committee of the University Hospital Split. Gestational age was determined based on external measurements and menstrual history. Tissue samples were classified into developmental phases according to a previously established classification based on specific developmental structures [24,25]. Phase 2 spans approximately 15 to 22 gestational weeks, corresponding to early metanephric development with active ureteric bud branching. Phase 3 covers approximately 22 to 36 gestational weeks, encompassing the period of active nephrogenesis. Phase 4 begins at approximately 36 gestational weeks and extends through the perinatal period, representing the completion of nephrogenesis and onset of nephron maturation. Phase 1 (weeks 5–14) was not included in the analysis due to the practical difficulty of obtaining samples at this early developmental stage. Of the 43 samples analyzed, 22 were normal control kidneys (CTRL), 2 were duplex kidneys (DK), 4 were horseshoe kidneys (HK), 3 were hypoplastic kidneys (HYP), and 12 were dysplastic kidneys (DYS). By developmental phase, the distribution was: Phase 2 (*n* = 16), Phase 3 (*n* = 21), and Phase 4 (*n* = 6). When stratified by both phenotype and developmental phase: CTRL Ph2/Ph3/Ph4 (*n* = 10/9/3), DK Ph2/Ph3/Ph4 (*n* = 0/2/0), HK Ph2/Ph3/Ph4 (*n* = 1/3/0), HYP Ph2/Ph3/Ph4 (*n* = 0/2/1), and DYS Ph2/Ph3/Ph4 (*n* = 5/5/2). Kidney pathology was assessed through morphological examination and standard histopathological methods. The kidney tissues were fixed in 4% paraformaldehyde, segmented into blocks, dehydrated through a graded alcohol series, and embedded in paraffin. Subsequently, tissue sections were cut by a microtome (5 μm).

### 2.2. Immunofluorescence

Paraffin-embedded tissue sections were initially deparaffinized with xylene (Sigma-Aldrich, St. Louis, MO, USA) and rehydrated through a graded alcohol series. Antigen retrieval was performed by heating the sections in 0.01 M citrate buffer (Sigma-Aldrich, St. Louis, MO, USA) at pH 6.0. Following three washes in phosphate-buffered saline (PBS)(Sigma-Aldrich, St. Louis, MO, USA), the sections were incubated with a blocking solution (ab64226, Abcam, Cambridge, UK) for 20 min. After removal of the blocking solution, primary antibodies were applied and incubated overnight in a humidified chamber. The sections were then washed in PBS, incubated with secondary antibodies for one hour at room temperature, and washed three times in PBS. Nuclear staining was conducted using DAPI (4′,6-diamidino-2-phenylindole, Sigma-Aldrich, St. Louis, MO, USA). Finally, the tissue sections were mounted with coverslips using Immuno-Mount medium (Thermo Shandon, Pittsburgh, PA, USA). Negative controls were prepared by omitting the primary antibody to confirm staining specificity. The antibodies utilized in this study are detailed in Table 2.

### 2.3. Data Acquisition and Image Analysis

Microphotographs were captured using a fluorescence microscope (Olympus BX51, Tokyo, Japan) with a Nikon DS-Ri2 camera (Nikon Corporation, Tokyo, Japan) and NIS-Elements F software (version 5.22.00). At an objective magnification of 40×, with standardized exposure times, a minimum of 20 non-overlapping fields per sample were randomly imaged to assess positive staining of ERK1/2, p38 MAPK, and JNK. In control kidneys, fields were selected from both the cortex and medulla. The distinctive structural abnormalities present in kidneys affected by CAKUT rendered differentiation between the cortex and medulla difficult in certain phenotypes, especially in dysplastic kidneys (DYS); therefore, fields were selected from representative tissue areas across the entire section. All analysts were blinded to sample group allocation during image analysis. Positive signals were identified by green fluorescence. Quantitative analysis involved calculating the percentage of the area exhibiting positive staining on each microphotograph. Representative images were assembled using Adobe Photoshop (version 25.0, Adobe Inc., San Jose, CA, USA). Captured images were analysed using ImageJ software (version 1.54, NIH, Bethesda, MD, USA) following established protocols as described in previous publications [26,27]. Briefly, fluorescence overlap was minimized by subtracting the red channel from the green fluorescence. A median filter with a 5.0-pixel radius was applied to duplicated images, and positive signals were extracted by subtracting the filtered images from the originals. Processed images were converted to 8-bit and modified using the triangle thresholding algorithm. The fluorescence area percentage was then calculated using the “Analyze Particles” tool. To address observer variability, three expert histologists independently analyzed the microphotographs and set thresholds using negative control images. Consistency was confirmed with an intraclass correlation coefficient above 0.8. All analysts were blinded to sample group allocation during image analysis.

### 2.4. Statistical Analysis

All statistical analyses were performed using GraphPad Prism software (version 10.6.1, GraphPad Software, La Jolla, CA, USA), with statistical significance set at *p* < 0.05. Data distribution was assessed using the Shapiro–Wilk normality test prior to selecting the appropriate statistical method for each comparison.

For comparisons of protein expression across kidney phenotypes (control, horseshoe, hypoplastic, and dysplastic kidneys), normally distributed data were analyzed using one-way ANOVA followed by Tukey’s multiple comparison test (ERK1/2, JNK), while non-normally distributed data were analyzed using the Kruskal–Wallis test followed by Dunn’s multiple comparison test (p38). Data for these analyses are presented as median with interquartile range. For comparisons across developmental phases (phases 2, 3, and 4), the Kruskal–Wallis test with Dunn’s post hoc test was applied for ERK1/2, JNK, and p38, as these data did not follow a normal distribution, and are likewise presented as median with interquartile range.

To evaluate the combined effects of phenotype and developmental phase on protein expression, two-way ANOVA followed by Tukey’s multiple comparison test was performed for ERK1/2, JNK, and p38, with phenotype and developmental phase as the two independent factors. Data for these analyses are presented as mean ± standard deviation (SD). Graphical representations were generated in GraphPad Prism.

## 3. Results

### 3.1. ERK1/2 Expression Is Reduced in Hypoplastic Human Fetal Kidney Tissue and Increases in Late Developmental Phase

**Figure 1:**
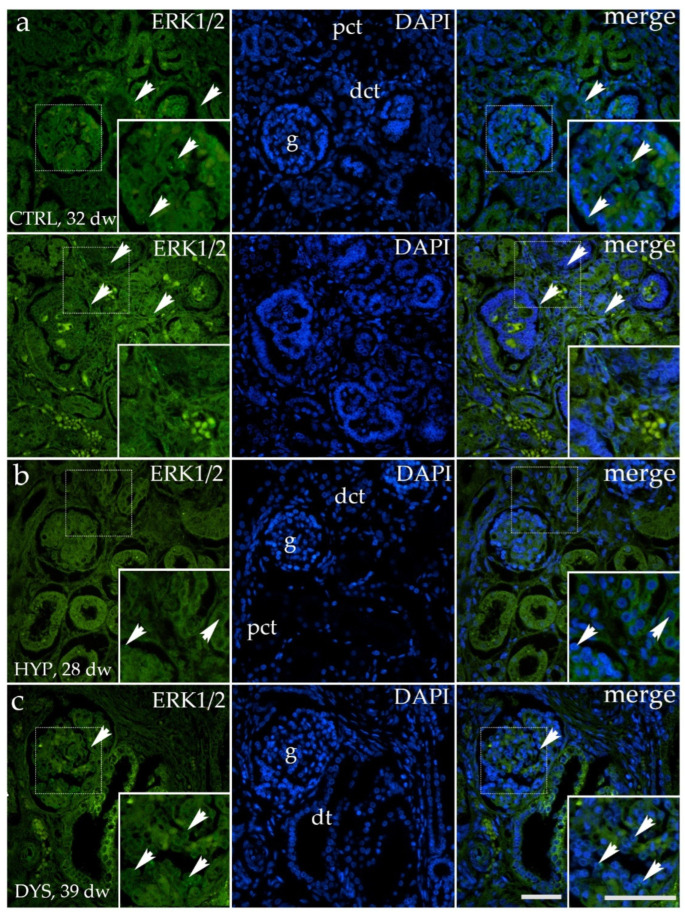
Immunofluorescence localization of ERK1/2 in human fetal kidney tissue. Representative immunofluorescence images showing ERK1/2 expression (green) in control (CTRL), hypoplastic (HYP), and dysplastic (DYS) human fetal kidney tissue at different developmental stages (control kidney, 32 developmental weeks; hypoplastic kidney, 28 developmental weeks; dysplastic kidney, 39 developmental weeks). Nuclei are counterstained with DAPI (blue), and merged images illustrate the spatial distribution of ERK1/2 immunoreactivity. ERK1/2 is predominantly localized in proximal and distal convoluted tubules (pct, dct), with additional staining in glomerular structures (g) and dysplastic tubules (dt). All images were taken at 40× magnification with a scale bar of 60 µm. White arrowheads indicate areas of prominent immunoreactivity.

Immunofluorescence analysis demonstrated a distinct spatial distribution of ERK1/2 expression in human fetal kidney tissue across different phenotypes and developmental stages (Figure 1). ERK1/2 immunoreactivity was predominantly localized to tubular compartments, including proximal and distal tubular segments, with additional staining observed in glomerular structures. The signal was mainly cytoplasmic within tubular epithelial cells and showed regional heterogeneity in intensity. ERK1/2 expression was detectable in both morphologically preserved and structurally altered renal tissue, providing a qualitative framework for subsequent quantitative analyses.

**Figure 2:**
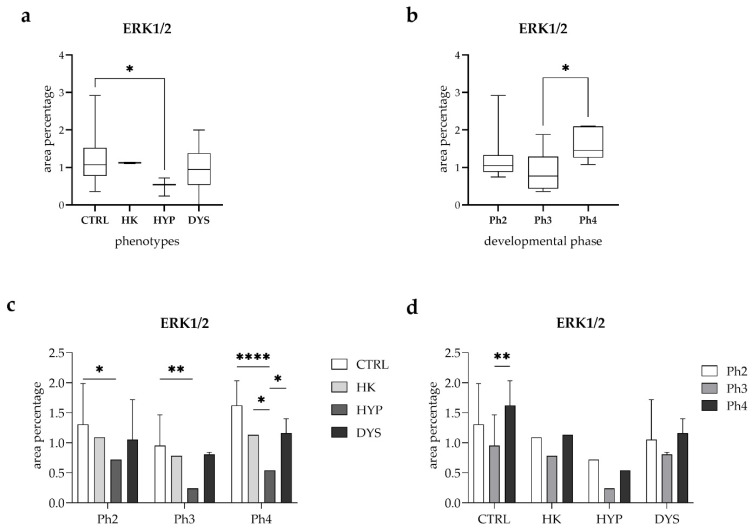
Quantitative analysis of ERK1/2 expression in human fetal kidney tissue. Box-and-whisker plots showing the quantitative analysis of ERK1/2 area percentage in control (CTRL), horseshoe kidney (HK), hypoplastic (HYP), and dysplastic (DYS) human fetal kidney tissue according to phenotype and developmental phase. ERK1/2 expression was analyzed across phenotypes (**a**), developmental phases (**b**), combined phenotype and developmental phase (**c**), and developmental phases within each phenotype (**d**). Data are presented as median with interquartile range (box-and-whisker plots, (**a**,**b**)) or mean ± standard deviation (bar graphs, (**c**,**d**)). Statistical significance was determined using appropriate post hoc tests. Asterisks indicate statistically significant differences (* *p* < 0.05, ** *p* < 0.01,**** *p* < 0.0001).

Quantitative assessment of ERK1/2 area percentage according to phenotype revealed no statistically significant difference between the CTRL and HK (horseshoe kidney) groups (Figure 2a). Although the CTRL group exhibited slightly higher values, this difference did not reach statistical significance. In contrast, ERK1/2 area percentage was significantly lower in the HYP (hypoplasia) group compared with CTRL (*p* < 0.05). No statistically significant difference was observed between the CTRL and DYS (dysplasia) groups.

When ERK1/2 expression was analyzed according to developmental phase (Figure 2b), no statistically significant differences were detected between phase 2 (Ph2) and phase 3 (Ph3), nor between phase 2 (Ph2) and phase 4 (Ph4). However, ERK1/2 area percentage was significantly higher in phase 4 (Ph4) compared with phase 3 (Ph3) (*p* < 0.05), with Ph4 showing the highest values among the analyzed developmental phases.

A combined analysis of phenotype and developmental phase further refined these observations (Figure 2c). Within phase 2 (Ph2), ERK1/2 area percentage did not differ significantly between CTRL and HK, CTRL and DYS, or among HK, HYP, and DYS groups. A significant reduction in ERK1/2 expression was observed in the HYP group compared with CTRL (*p* < 0.05), while all other pairwise comparisons showed no statistically significant differences.

In phase 3 (Ph3), ERK1/2 area percentage remained comparable between CTRL and HK, CTRL and DYS, and among HK, HYP, and DYS groups. However, ERK1/2 expression was significantly lower in the HYP group compared with CTRL (*p* < 0.01). In phase 4 (Ph4), ERK1/2 area percentage did not differ significantly between CTRL and HK or between CTRL and DYS. A marked reduction in ERK1/2 expression was observed in the HYP group compared with CTRL (*p* < 0.0001). Additionally, ERK1/2 area percentage was significantly higher in the HK group compared with HYP (*p* < 0.05), as well as higher in the DYS group compared with HYP (*p* < 0.05), while no significant difference was detected between HK and DYS.

Finally, developmental phase–dependent changes in ERK1/2 expression were analyzed within each phenotype (Figure 2d). Within the CTRL group, no statistically significant differences were observed between Ph2 and Ph3 or between Ph2 and Ph4; however, ERK1/2 area percentage was significantly higher in Ph4 compared with Ph3 (*p* < 0.01). In contrast, no statistically significant differences between developmental phases were detected within the HK, HYP, or DYS groups.

### 3.2. p38 Expression Is Largely Stable Across Phenotypes, with Phase-Specific Alterations in Human Fetal Kidney

**Figure 3:**
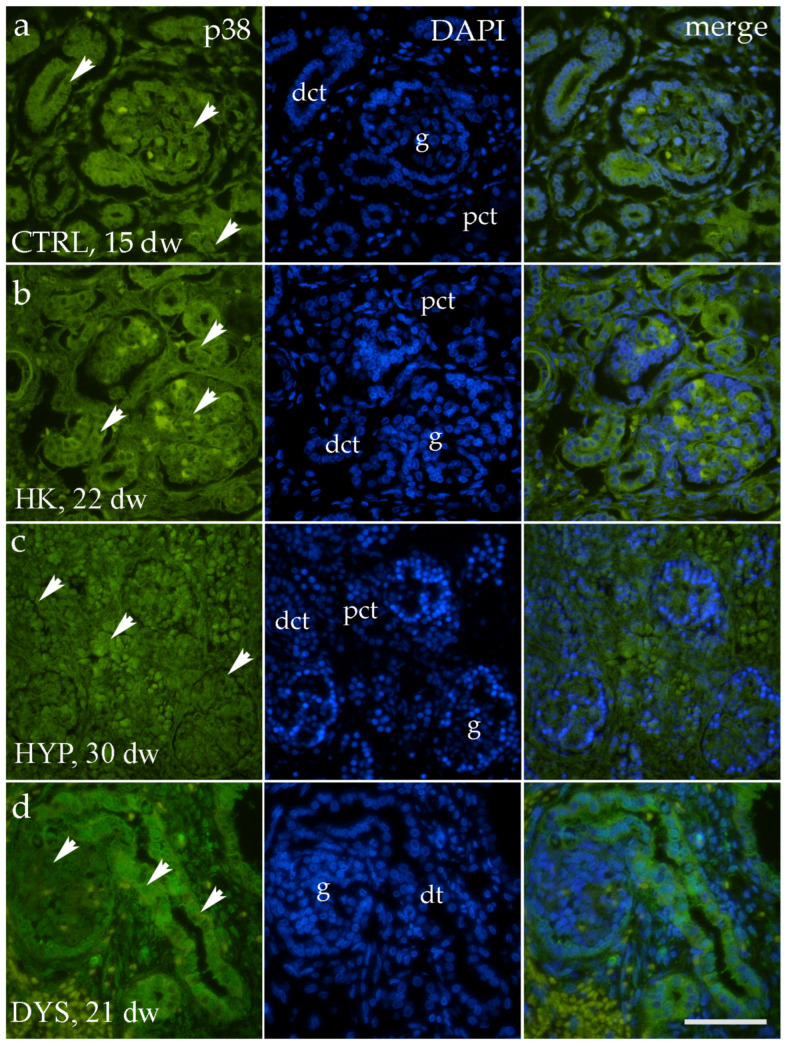
Immunofluorescence localization of p38 in human fetal kidney tissue. Representative immunofluorescence images showing p38 expression (green) in control (CTRL), hypoplastic (HYP), and dysplastic (DYS) human fetal kidney tissue at different developmental stages (control kidney, 32 developmental weeks; hypoplastic kidney, 28 developmental weeks; dysplastic kidney, 39 developmental weeks). Nuclei are counterstained with DAPI (blue), and merged images illustrate the spatial distribution of p38 immunoreactivity. p38 is predominantly localized in proximal and distal convoluted tubules (pct, dct), with additional staining in glomerular structures (g) and dysplastic tubules (dt). All images were taken at 40× magnification with a scale bar of 60 µm. White arrowheads indicate areas of prominent immunoreactivity.

Immunofluorescence analysis revealed the presence of p38 immunoreactivity in human fetal kidney tissue across different phenotypes and developmental stages (Figure 3). p38 expression was predominantly localized to tubular compartments, including proximal and distal convoluted tubules, with additional staining observed in glomerular structures. The signal was mainly cytoplasmic within tubular epithelial cells and showed regional heterogeneity in intensity. In dysplastic kidneys, p38 immunoreactivity was also evident within dysplastic tubular structures. These qualitative observations provided a morphological framework for subsequent quantitative analyses.

**Figure 4:**
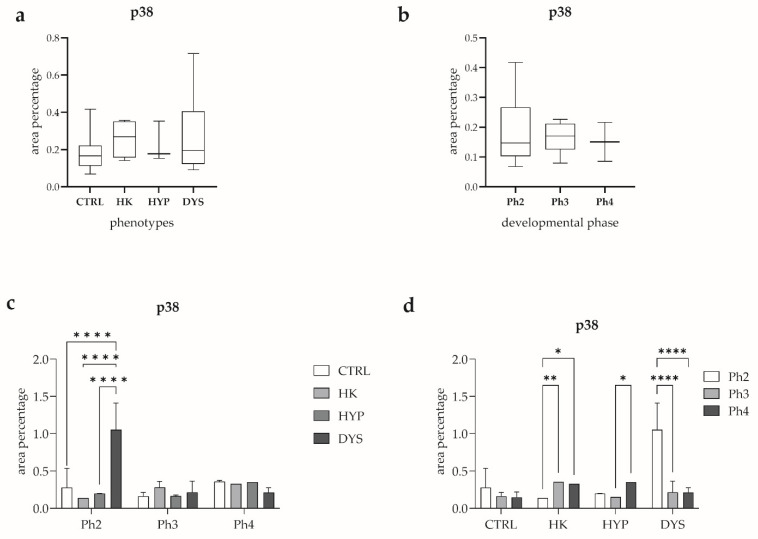
Quantitative analysis of p38 expression in human fetal kidney tissue. Box-and-whisker plots showing the quantitative analysis of p38 area percentage in control (CTRL), horseshoe kidney (HK), hypoplastic (HYP), and dysplastic (DYS) human fetal kidney tissue according to phenotype and developmental phase. p38 expression was analyzed across phenotypes (**a**), developmental phases (**b**), combined phenotype and developmental phase (**c**), and developmental phases within each phenotype (**d**). Data are presented as median with interquartile range (box-and-whisker plots, (**a**,**b**)) or mean ± standard deviation (bar graphs, (**c**,**d**)). Statistical significance was determined using appropriate post hoc tests. Asterisks indicate statistically significant differences (* *p* < 0.05, ** *p* < 0.01, **** *p* < 0.0001).

Quantitative assessment of p38 area percentage according to phenotype showed no statistically significant differences among the examined groups (Figure 4a). Specifically, p38 expression did not differ significantly between the CTRL and HK (horseshoe kidney) groups, between CTRL and HYP (hypoplasia), or between CTRL and DYS (dysplasia).

Similarly, when p38 area percentage was analyzed according to developmental phase alone (Figure 4b), no statistically significant differences were observed between phase 2 (Ph2) and phase 3 (Ph3), between Ph2 and phase 4 (Ph4), or between Ph3 and Ph4.

A combined analysis of phenotype and developmental phase revealed phase- and phenotype-specific differences in p38 expression (Figure 4c). Within phase 2 (Ph2), p38 area percentage did not differ significantly between CTRL and HK or between CTRL and HYP. In contrast, p38 expression was significantly higher in the DYS group compared with CTRL, HK, and HYP (*p* < 0.0001 for all comparisons). No statistically significant difference was detected between the HK and HYP groups. In phase 3 (Ph3), no statistically significant differences in p38 area percentage were observed between any of the analyzed phenotypes. Likewise, in phase 4 (Ph4), p38 expression remained comparable across all phenotypic groups, with no statistically significant differences detected.

Finally, developmental phase–dependent changes in p38 expression were analyzed within each phenotype (Figure 4d). Within the CTRL group, no statistically significant differences were observed between Ph2 and Ph3, Ph2 and Ph4, or between Ph3 and Ph4. In contrast, within the HK group, p38 area percentage was significantly higher in Ph3 compared with Ph2 (*p* < 0.01) and higher in Ph4 compared with Ph2 (*p* < 0.05), while no significant difference was observed between Ph3 and Ph4. Within the HYP group, p38 expression did not differ significantly between Ph2 and Ph3 or between Ph2 and Ph4; however, p38 area percentage was significantly higher in Ph4 compared with Ph3 (*p* < 0.05). Within the DYS group, p38 area percentage was significantly higher in Ph2 compared with both Ph3 and Ph4 (*p* < 0.0001 for both comparisons), whereas no statistically significant difference was observed between Ph3 and Ph4.

### 3.3. JNK Expression Is Selectively Increased in Horseshoe Kidney in a Developmental Phase–Dependent Manner

**Figure 5:**
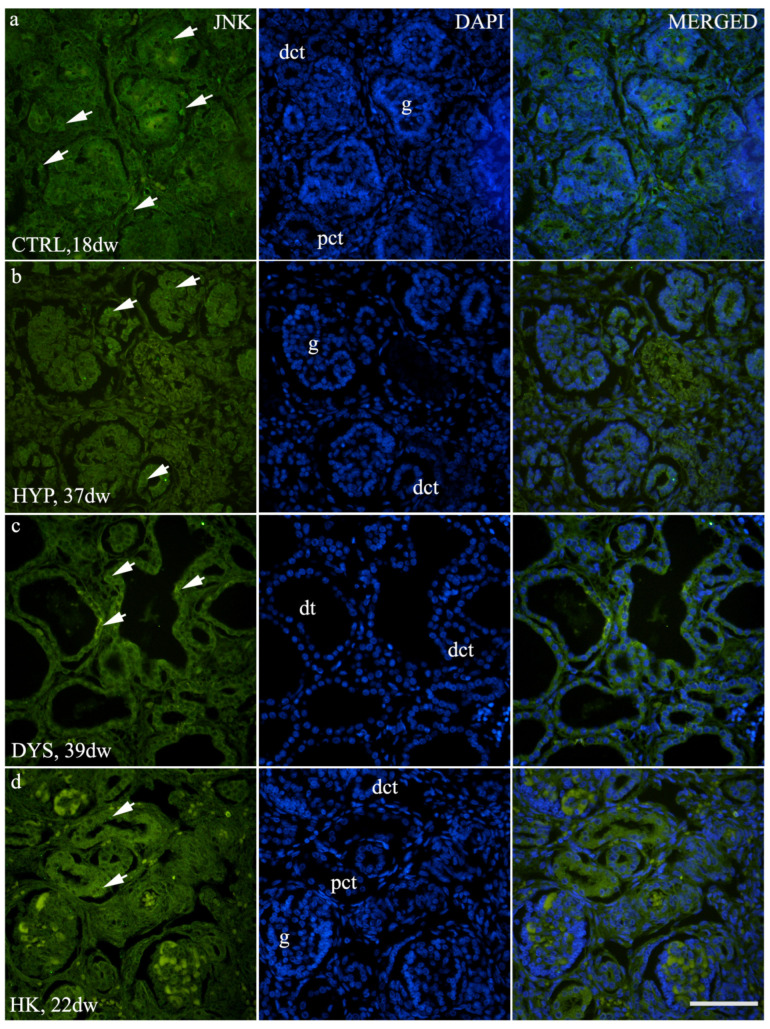
Immunofluorescence localization of JNK in human fetal kidney tissue. Representative immunofluorescence images showing JNK expression (green) in control (CTRL), hypoplastic (HYP), and dysplastic (DYS) human fetal kidney tissue at different developmental stages (control kidney, 32 developmental weeks; hypoplastic kidney, 28 developmental weeks; dysplastic kidney, 39 developmental weeks). Nuclei are counterstained with DAPI (blue), and merged images illustrate the spatial distribution of JNK immunoreactivity. JNK is predominantly localized in proximal and distal convoluted tubules (pct, dct), with additional staining in glomerular structures (g) and dysplastic tubules (dt). All images were taken at 40× magnification with a scale bar of 60 µm. White arrowheads indicate areas of prominent immunoreactivity.

Immunofluorescence analysis demonstrated detectable JNK expression in human fetal kidney tissue across different phenotypes and developmental stages (Figure 5). JNK immunoreactivity was predominantly localized within tubular compartments, including proximal and distal convoluted tubules, with additional staining observed in glomerular structures. The signal was mainly cytoplasmic within tubular epithelial cells and showed regional heterogeneity in intensity. JNK expression was evident in control, horseshoe kidney, hypoplastic, and dysplastic fetal kidneys, including within dysplastic tubular structures, providing a qualitative framework for subsequent quantitative analyses.

**Figure 6:**
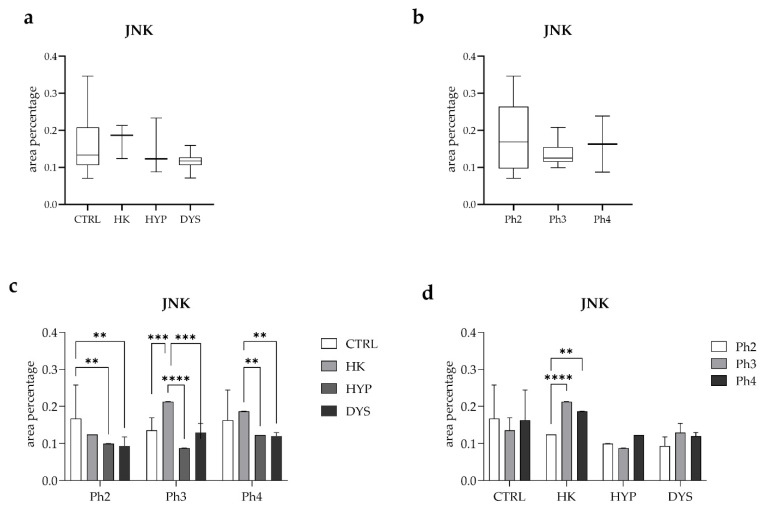
Quantitative analysis of JNK expression in human fetal kidney tissue. Box-and-whisker plots showing the quantitative analysis of JNK area percentage in control (CTRL), horseshoe kidney (HK), hypoplastic (HYP), and dysplastic (DYS) human fetal kidney tissue according to phenotype and developmental phase. JNK expression was analyzed across phenotypes (**a**), developmental phases (**b**), combined phenotype and developmental phase (**c**), and developmental phases within each phenotype (**d**). Data are presented as median with interquartile range (box-and-whisker plots, (**a**,**b**)) or mean ± standard deviation (bar graphs, (**c**,**d**)). Statistical significance was determined using appropriate post hoc tests. Asterisks indicate statistically significant differences (** *p* < 0.01, *** *p* < 0.001, **** *p* < 0.0001).

Quantitative assessment of JNK area percentage according to phenotype revealed no statistically significant differences among the examined groups (Figure 6a). Specifically, JNK expression did not differ significantly between the CTRL and HK (horseshoe kidney) groups, between CTRL and HYP (hypoplasia), or between CTRL and DYS (dysplasia). Likewise, analysis of JNK area percentage according to developmental phase alone showed no statistically significant differences between phase 2 (Ph2) and phase 3 (Ph3), between Ph2 and phase 4 (Ph4), or between Ph3 and Ph4 (Figure 6b).

A combined analysis of phenotype and developmental phase revealed phase- and phenotype-specific differences in JNK expression (Figure 6c). Within phase 2 (Ph2), JNK area percentage did not differ significantly between CTRL and HK. However, JNK expression was significantly higher in the CTRL group compared with both the HYP (*p* < 0.01) and DYS (*p* < 0.01) groups, while no statistically significant differences were observed between HK and HYP, HK and DYS, or between HYP and DYS. In phase 3 (Ph3), JNK area percentage was significantly higher in the HK group compared with CTRL (*p* < 0.001). No statistically significant differences were detected between CTRL and HYP or between CTRL and DYS. Additionally, JNK expression was significantly higher in the HK group compared with both HYP (*p* < 0.0001) and DYS (*p* < 0.001), whereas no significant difference was observed between HYP and DYS. In phase 4 (Ph4), no statistically significant differences were observed between CTRL and HK, CTRL and HYP, or between CTRL and DYS. In contrast, JNK area percentage was significantly higher in the HK group compared with both the HYP (*p* < 0.01) and DYS (*p* < 0.01) groups, while no statistically significant difference was detected between HYP and DYS.

Finally, developmental phase–dependent changes in JNK expression were analyzed within each phenotype (Figure 6d). Within the CTRL group, no statistically significant differences were observed between Ph2 and Ph3, Ph2 and Ph4, or between Ph3 and Ph4. In contrast, within the HK group, JNK area percentage was significantly higher in Ph3 compared with Ph2 (*p* < 0.0001) and higher in Ph4 compared with Ph2 (*p* < 0.01), while no significant difference was observed between Ph3 and Ph4. Within the HYP and DYS groups, no statistically significant differences in JNK expression were detected between any of the examined developmental phases.

## 4. Discussion

Mitogen-activated protein kinases (MAPKs) are key intracellular signalling molecules that regulate cellular proliferation, differentiation, and apoptosis [7,8,19]. This study examined ERK1/2, p38, and JNK expression patterns in human fetal kidney tissue during normal development and in congenital anomalies: control (CTRL), horseshoe kidney (HK), hypoplasia (HYP), and dysplasia (DYS) phenotypes at various developmental phases.

ERK1/2 is essential for ureteric bud branching morphogenesis, nephron progenitor maintenance, and differentiation. In control kidneys, ERK1/2 was localised to tubular compartments and glomerular structures, with progressive upregulation during Phase 4, consistent with the critical transition from renal vesicles to mature nephron segments [6,20]. Hypoplastic kidneys exhibited significant ERK1/2 reduction across all phases, with loss of the normal Phase 3–4 upregulation. This finding aligns coherently with the defining pathology of renal hypoplasia: reduced nephron endowment resulting from impaired nephrogenic progenitor expansion and premature exhaustion of the nephrogenic zone. Since ERK1/2 signalling is required for sustaining nephron progenitor proliferation and preventing their premature differentiation or depletion [20,21,22], its reduction across all developmental phases likely reflects a primary failure to maintain adequate progenitor pool dynamics, ultimately resulting in the reduced nephron number characteristic of hypoplastic kidneys [6,7,28]. Dysplastic and horseshoe kidneys exhibited ERK1/2 levels comparable to controls, in contrast to Omori’s report of ectopic ERK phosphorylation in dysplasia. This discrepancy likely reflects differences in measuring total protein versus phosphorylation status. As a horseshoe kidney results from structural fusion during early development rather than primary differentiation defects, preserved ERK1/2 patterns suggest that nephrogenic programmes remain intact despite the morphological abnormality [23,29,30,31].

p38 expression showed no significant phenotypic differences overall, in contrast to pronounced ERK1/2 variations. However, within the horseshoe kidney phenotype, p38 was significantly elevated in Phases 3–4 compared with Phase 2, suggesting compensatory stress-response activation triggered by structural fusion, abnormal vascularization, and mechanical constraints [32,33,34]. This selective upregulation in the horseshoe kidney, specifically absent in hypoplasia and dysplasia, supports the interpretation that p38 responds to structural/mechanical perturbations rather than primary differentiation failure [31,35]. Our observation of stable total p38 protein contrasts with Omori’s finding of ectopic p38 phosphorylation in dysplastic kidney epithelia, where both p38 and phospho-p38 are strongly expressed but notably absent in normal kidneys at any developmental stage [31,36]. This fundamental difference, stable total protein with late-phase elevation in the horseshoe kidney versus ectopic expression with maximal Phase 2 activation in dysplasia, underscores distinct pathogenic mechanisms. The temporal pattern in dysplastic kidneys, with maximal p38 expression in Phase 2 followed by diminishing levels in later phases [31], suggests an early stress response to the initiation of dysmorphogenesis, followed by loss of dynamic regulation as undifferentiated progenitors fail to progress. In contrast, our finding of late-phase (Phases 3–4) p38 elevation in the horseshoe kidney is consistent with mechanical stress-induced activation, as cyclic stretch has been shown to markedly activate p38 in renal cells in a pressure-dependent manner [35,37]. Furthermore, Hida et al. demonstrated that p38 is required for kidney growth and nephrogenesis during normal development [6], supporting our interpretation that late-phase p38 upregulation in the horseshoe kidney may represent an adaptive compensatory mechanism responding to fusion-imposed mechanical constraints and altered hemodynamics, rather than the primary dysregulatory event seen in dysplasia. Dysplasia involves sustained ERK1/2 and ectopic p38 activation with disrupted epithelial-mesenchymal interactions: dysplastic progenitors retain ERK1/2 signaling but cannot respond to upstream inductive signals required for normal tubulogenesis [29,31,38].

When examined separately or by phase alone, JNK did not show any phenotypic variations. However, during Phase 4, phenotype-phase analysis revealed that JNK was selectively elevated in the horseshoe kidney. Stress signals, including mechanical, oxidative, and inflammatory stimuli, preferentially activate JNK rather than growth factor signalling such as ERK1/2 [8,14,39,40]. The late-phase JNK increase in the horseshoe kidney most likely reflects the activation of stress-response pathways triggered by structural constraints imposed by fusion, altered blood flow, and oxygen tension during the completion of nephrogenesis. Our findings differ from those of Omori et al., who reported JNK downregulation in dysplastic kidney epithelia [31]. This difference may reflect fundamental distinctions between dysplastic and fusion anomalies: in dysplasia, JNK downregulation is accompanied by ectopic p38 activation and sustained ERK1/2 signalling in undifferentiated epithelia [31], whereas in the horseshoe kidney, late-phase JNK elevation likely represents an adaptive stress response to mechanical constraints and altered haemodynamics specific to fusion anomalies. In contrast, BMP7-mediated JNK activation promotes nephron progenitor proliferation during normal nephrogenesis [23,32,40], suggesting that the temporal JNK increase observed in Phase 4 may support compensatory progenitor expansion in response to developmental stress imposed by fusion.

Hypoplasia exhibits the most pronounced dysregulation, with persistent ERK1/2 reduction across all phases and a lack of Phase 3–4 upregulation, suggesting a fundamental uncoupling of proliferation-to-differentiation signals, possibly due to a shift towards alternative proliferative pathways. Horseshoe kidney shows preserved ERK1/2 patterns with normal phase-dependent maturation, indicating intact nephrogenic programmes; however, selective p38/JNK upregulation during Phases 3–4 represents compensatory stress-response activation to structural deformity. This explains why a horseshoe kidney often retains adequate renal function despite anatomical abnormality, whereas dysplasia and hypoplasia frequently result in nephropathy [7,41,42,43].

These distinct MAPK expression patterns across CAKUT phenotypes suggest potential diagnostic biomarkers for differentiating congenital kidney anomalies and predicting functional outcomes. Therapeutic approaches should be phenotype-specific: restoring differentiation-permissive ERK1/2 signalling in hypoplasia, targeting disrupted epithelial-mesenchymal interactions in dysplasia, and managing stress pathway activation in the horseshoe kidney. Future studies should use phospho-kinase-specific antibodies, examine cross-talk with developmental signalling cascades (Notch, WNT, BMP), and employ organ culture systems with selective kinase inhibition to establish causative relationships.

This study has several limitations that should be acknowledged. The sample size for certain CAKUT subtypes is relatively small, which is inherent to the nature of the material, human fetal kidney tissue is exceptionally rare and difficult to obtain, making these specimens of considerable scientific value. The use of archived formalin-fixed paraffin-embedded tissue, while standard for this type of study, restricted the application of additional protein quantification methods. Furthermore, this study assessed total protein expression rather than the phosphorylated forms of ERK1/2, p38, and JNK; future studies employing phospho-specific antibodies would provide complementary insights into pathway activation dynamics. Finally, the absence of Phase 1 samples limits analysis of the earliest stages of metanephric development.

## 5. Conclusions

This study demonstrates that ERK1/2, p38, and JNK exhibit distinct developmental and phenotype-specific expression patterns in human fetal kidneys. ERK1/2 expression progressively increases during late nephrogenesis in control kidneys but is significantly reduced across all developmental phases in hypoplastic kidneys, consistent with impaired progenitor pool maintenance and reduced nephron endowment. p38 and JNK show selective phase-dependent upregulation in horseshoe kidneys, reflecting compensatory stress-response activation driven by structural fusion and mechanical constraints, rather than primary differentiation failure. Dysplastic kidneys display an early p38 stress response that diminishes over time, suggesting a distinct pathogenic mechanism characterized by early dysmorphogenesis and failure of progenitor progression. These divergent MAPK expression profiles across CAKUT phenotypes highlight the molecular heterogeneity of congenital renal anomalies and may contribute to improved phenotype-specific characterization. Future studies employing phospho-specific antibodies and functional kinase inhibition models will be essential to establish causative relationships and explore the therapeutic potential of targeting these pathways in CAKUT.

## Figures and Tables

**Table 1:** Human fetal kidney samples analyzed in the study (No. 43).

Groups	Phase of Development	Renal Pathology and Associated Conditions	Weeks of Gestation	No.
Normal kidneys (CTRL)	Phase 2	No	15	1
		No	15–16	2
		No	16	1
		No	16–17	1
		No	18	1
		No	18	2
		No	21	2
	Phase 3	No	24	2
		No	28	2
		No	28	1
		No	32	2
		No	35	2
	Phase 4	No	37	2
		No	38	1
Horseshoe kidneys (HK)	*Ren concreatus arcuatus, cystae multiplices corticales*		22	1
	*Ren concretus arcuatus, tetralogija Fallot*		26	1
	*Syndroma Edwards, Ren arcuatus*		30–31	1
	*Syndroma Edwards, Ren arcuatus*		34 + 4 days	1
Hypoplastic kidneys (HYP)	*Hypoplasia renis*		28	1
	*Hypoplasia renis lateris dextri*		30 + 5 days	1
	*Hypoplasia renis sinister*		37 + 5 days	1
Dysplastic kidneys (DYS)	*Megaureter lateris dextri, dysplasia renalis*		21	1
	*Agenesia renis dextri, ren unilateralis*		21	2
	*Ren sinister cysticus, ren dexter absens*		21–22	2
	*Dysplasia multicysticarenis dextri/Cystes parvae focales*		27	2
	*Dysplasia Hypoplastica*		33–34	1
	*Renes dysplastici cystici, Potter syndroma*		35	2
	*Agenesi renis dextri et dysplasia renis sinistri cum ureter duplex, Currarino syndrome*		37	1
	*Dysplasia Hypoplastica, renalis bilateralis Down, Potter syndrome*		38	1

**Table 2:** List of antibodies used in the study (primary and secondary).

Antibodies		Catalog No.	Host	Dilution	Source
Primary	p44/42 MAPK (Erk1/2) (137F5)	4695S	Rabbit	1:300	Cell Signaling Technology, Inc. (CST) Danvers, MA, USA
	p38 MAPK Antibody	9212S	Rabbit	1:100	
	SAPK/JNK Antibody	9252S	Rabbit	1:100	
Secondary	Alexa Fluor^®^ 488 AffiniPure^®^ Donkey Anti-Rabbit IgG (H + L)	711-545-152	Donkey	1:300	Jackson ImmunoResearch Laboratories, Inc., West Grove, PA, USA

## Data Availability

The original contributions of this study are fully incorporated within the published article. Any additional questions or requests for information may be directed to the corresponding author for further clarification.
